# Coding-complete genome sequence of an *Alphacoronavirus* isolated in *Rattus norvegicus* captured in an urban park in France

**DOI:** 10.1128/mra.00230-25

**Published:** 2025-05-30

**Authors:** Kevyn Beissat, Evelyne Picard-Meyer, Virginie Lattard, Fanny Bastien, Jean-Luc Schereffer, Sionfoungo Daouda Soro, Elodie Monchâtre-Leroy, Marine Wasniewski

**Affiliations:** 1 Nancy Laboratory for Rabies and Wildlife, ANSES, Malzéville, Alsace-Champagne-Ardenne-Lorraine, France; 2 USC-1233 Rongeurs Sauvages Risques Sanitaires et Gestion des Populations (RS2GP), VetAgroSup, Institut National de Recherche pour l’Agriculture, l’Alimentation et l’Environnement (INRAE), Lyon University, Marcy-L'Etoile, France; DOE Joint Genome Institute, Berkeley, California, USA

**Keywords:** *alphacoronavirus*, *Rattus norvegicus*, complete genome, France

## Abstract

Here, we described an *Alphacoronavirus* coding-complete genome identified in a French brown rat. This *Alphacoronavirus* was detected in *Rattus norvegicus* intestines (colon) collected in 2022 in Marseille, France.

## ANNOUNCEMENT

Coronaviruses are members of the *Coronaviridae* family and are classified into four recognized genera: *Alphacoronavirus*, *Betacoronavirus*, *Gammacoronavirus*, and *Deltacoronavirus*. The *Alphacoronavirus* genus is further subdivided into 15 subgenera, including the *Luchacovirus*, which is exclusively associated with rodent coronaviruses (1). Alphacoronaviruses have been detected in *Rattus* spp. captured in several countries (2–9). Furthermore, two human coronaviruses, HCoV-HKU1 and HCoV-OC43, have been shown to have originated from murine sources (10). This underscores the significance of understanding the coronaviruses that are currently circulating among rodents. In this study, we present the detection of an *Alphacoronavirus* in a *Rattus norvegicus* trapped in 2022 in France. For other European countries, only RNA-dependent RNA polymerase (RdRp) or ORF1ab sequences are currently accessible in public databases (2, 9).

In December 2022, a colon was collected from a *Rattus norvegicus* captured in an urban park (43.2748999°N, 5.3959662°E) in Marseille, Bouches-du-Rhône, France. The colon was stored dry at −80°C in a sterile tube. Rat capture and euthanasia procedures were approved by the Ethics Committee of the Veterinary School of Lyon and authorized by the French Ministry of Research (n°#37713-2022042712262465).

Total RNA was extracted from the colon using the NucleoSpin RNA Mini Kit (Macherey-Nagel). Subsequently, RT-PCR and nested PCR targeting the RdRp gene were performed using the following primers: PanCov Pol 15197-Forward: GGTTGGGACTATCCTAAGTGTGA and PanCov Pol 15635-Reverse: CCATCATCAGATAGAATCATCAT (11) are able to detect Alpha- and Betacoronaviruses.

The sample was subjected to next-generation sequencing analysis by Eurofins Genomics using the INVIEW Virus protocol (NEB Next Ultra Kit). The library was subjected to Illumina NovaSeq sequencing with 2 × 150 bp, resulting in 33M raw reads (22M unique reads). Quality controls and trimming were performed using MULTIQC (v1.27) (12) and Cutadapt (v4.6) (13), respectively, resulting in 13.5M trimmed reads. Sequences were then mapped to the KY370050 sequence using MAP with BWA-MEM (v0.7.18) (14) (41727 mapped reads). Alignments were checked using Integrative Genomics Viewer (v2.19.1) (15). The average depth of coverage was 207× and all regions were covered by reads. Finally, a consensus sequence was identified using IVAR CONSENSUS (v1.4.3) (16). Unless otherwise noted, default parameters were used.

**Fig 1:**
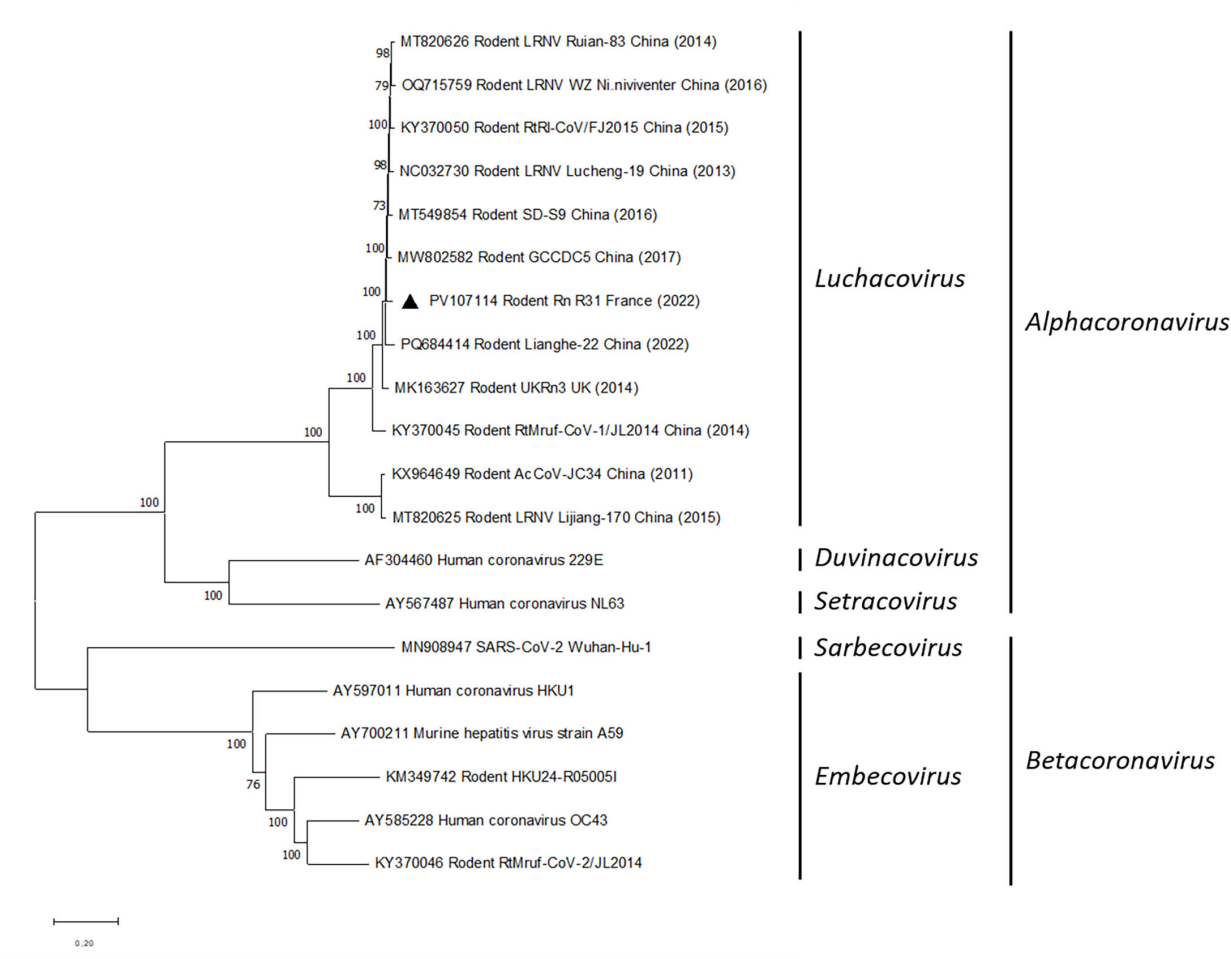
Phylogenetic tree of rodent coronaviruses constructed using the maximum likelihood method (GTR+G+I model) with MEGA version 11 (17). Eleven whole genomes of rodent alphacoronaviruses and PV107114 (black triangle) were aligned against eight referenced coronavirus sequences from ICTV, using MUSCLE (18). The bootstrap probabilities of each node were calculated using 1000 replicates. Bootstrap values were indicated at each branch.

The *Alphacoronavirus Rattus norvegicus* sequence presented here consisted of 28,665 bp, with a GC content of 40.4%. A preliminary analysis indicates that our sequence belongs to the *Luchacovirus* subgenus (Fig. 1). By BLASTn, our sequence exhibited 97.39% and 97.04% sequence identity, respectively, with two sequences isolated from a *Rattus norvegicus* in 2017 and 2013 in China (MW802582 and NC_032730). These findings demonstrate that *Rattus norvegicus* trapped in China and France harbored similar strains of *Alphacoronavirus*.

## Data Availability

The complete genome is deposited in GenBank under the accession number PV107114, and raw reads are available in SRA (SRR32311115).
